# Limiting collagen turnover via collagenase‐resistance attenuates right ventricular dysfunction and fibrosis in pulmonary arterial hypertension

**DOI:** 10.14814/phy2.12815

**Published:** 2016-06-01

**Authors:** Mark J. Golob, Zhijie Wang, Anthony J. Prostrollo, Timothy A. Hacker, Naomi C. Chesler

**Affiliations:** ^1^ Department of Biomedical EngineeringUniversity of Wisconsin‐Madison College of EngineeringMadisonWisconsin; ^2^ Materials Science ProgramUniversity of Wisconsin‐Madison College of EngineeringMadisonWisconsin; ^3^ Department of MedicineMedical Science CenterUniversity of Wisconsin‐MadisonMadisonWisconsin

**Keywords:** Cardiac energetics, effective arterial elastance, hypertrophy, pressure‐volume loop

## Abstract

Pulmonary arterial hypertension (PAH) is a severe form of pulmonary hypertension in which right ventricular (RV) afterload is increased and death typically occurs due to decompensated RV hypertrophy and failure. Collagen accumulation has been implicated in pulmonary artery remodeling, but how it affects RV performance remains unclear. Here, we sought to identify the role of collagen turnover, defined as the balance between collagen synthesis and degradation, in RV structure and function in PAH. To do so, we exposed mutant (Col1a1^R/R^) mice, in which collagen type I degradation is impaired such that collagen turnover is reduced, and wild‐type (Col1a1^+/+^) littermates to 14 days of chronic hypoxia combined with SUGEN treatment (HySu) to recapitulate characteristics of clinical PAH. RV structure and function were measured by echocardiography, RV catheterization, and histology. Despite comparable increases in RV systolic pressure (Col1a1^+/+^: 46 ± 2 mmHg; Col1a1^R/R^: 47 ± 3 mmHg), the impaired collagen degradation in Col1a1^R/R^ mice resulted in no RV collagen accumulation, limited RV hypertrophy, and maintained right ventricular‐pulmonary vascular coupling with HySu exposure. The preservation of cardiac function in the mutant mice indicates a beneficial role of limited collagen turnover via impaired degradation in RV remodeling in response to chronic pressure overload. Our results suggest novel treatments that reduce collagen turnover may offer a new therapeutic strategy for PAH patients.

## Introduction

Pulmonary arterial hypertension (PAH) is a severe pulmonary vascular disease in which increased right ventricular (RV) afterload leads to decompensated RV hypertrophy, failure, and ultimately death (McLaughlin et al. 2009). Accumulation of the extracellular matrix protein collagen, that is, fibrosis, is a key feature of the vascular remodeling that causes increased RV afterload. In particular, fibrosis causes proximal arterial stiffening (Ooi et al. 2010; Wang and Chesler 2012; Wang et al. 2013a) and may contribute to pulmonary vascular narrowing since circulating biomarkers of collagen metabolism predict disease severity in PAH patients (Safdar et al. 2014). The impact of fibrosis in the RV in PAH is less clear.

Recent evidence suggests that manipulating collagen turnover, that is, the balance between synthesis and degradation, is important in ventricular dysfunction (D'Armiento 2002). Collagen turnover can be altered by interfering with synthesis, degradation, or both. Limiting collagen degradation via matrix metalloproteinase (MMP) inhibition or deletion protects against myocardial infarction‐induced dilation of the left ventricle (LV) (Ducharme et al. 2000) and prevents the transition to a decompensated LV with pressure overload (Peterson et al. 2001). In the RV, increased collagen degradation via enhanced MMP activity causes systolic dysfunction (Baicu et al. 2003). However, the effect of reduced collagen turnover via impaired degradation on RV functional changes in PAH remains unclear. Here, we investigated the effect of limiting collagen turnover through impaired collagen degradation on RV function by using a transgenic mouse strain (Col1a1). A substitution in the Col1a1 gene in mutant (Col1a1^R/R^) mice results in a collagen type I triple helix that is resistant to collagenase‐based degradation (Wu et al. 1990; Liu et al. 1995).

To investigate the role of collagen turnover in RV structural and functional adaptation to increased afterload, RV function must be measured simultaneously with RV afterload. Quantifying the efficiency of ventricular–vascular interactions provides important information regarding cardiac function through energy transfer from the ventricles to the vasculature. Our group previously used these techniques to demonstrate that an agent that blocks collagen synthesis limits PAH progression and RV hypertrophy and fibrosis and maintains the efficiency of ventricular–vascular interactions in response to hypoxia‐induced PAH (Schreier et al. 2013). However, the functional and structural outcomes can be attributed to the reduced severity of PAH in this animal model since the increase in RV systolic pressure (RVSP) was mild. Here, we sought to test the hypothesis that collagen turnover via impaired collagen degradation during RV remodeling is a critical contributor to RV hypertrophy and dysfunction in severe PAH.

To test our hypothesis, severe PAH was created in Col1a1^R/R^ mice and wild‐type littermates (Col1a1^+/+^) using a combination of chronic hypoxia and SUGEN, a vascular endothelial growth factor receptor inhibitor, which recapitulates characteristics of human PAH (Ciuclan et al. 2011). Then, echocardiography, right heart catheterization, and histology were performed to quantify ventricular–vascular interactions and RV function and structure. Our results show that the severity of PAH was similar between the strains, which allowed us to study how impaired collagen turnover affects RV remodeling in PAH. We found that limiting collagen turnover through collagenase‐resistance attenuates RV hypertrophy and fibrosis and preserves RV function in PAH.

## Materials and Methods

### Animal handling

Eight‐week old male and female Col1a1^+/+^ (*n* = 7) and Col1a1^R/R^ (*n* = 10) mice were exposed to a combination of chronic hypoxia and SUGEN (HySu) for 14 days as previously described (Wang et al. 2013b). Preliminary studies found no differences in cardiac function between untreated wild‐type and mutant mice at 8 weeks of age (unpublished results). Prior results found no differences in pulmonary vascular structure and function between untreated wild‐type and mutant mice at 16–18 weeks of age (Wang et al. 2013a). The duration of exposure (14 days) was chosen to allow comparison to previous results (Wang et al. 2013c). Briefly, mice were exposed to normobaric hypoxia (10% O_2_) in a controlled chamber and given a weekly treatment of SUGEN (SU5416; Sigma‐Aldrich Corp., St. Louis, MO, intraperitoneal injection) at the dose of 20 mg/kg (Wang et al. 2013b). Administration of SUGEN causes a more severe form of PAH than hypoxia exposure alone (Ciuclan et al. 2011). The chamber was opened for less than 20 min at a time for regular animal care and SUGEN treatment. Levels of carbon dioxide, temperature, oxygen, and humidity were measured with a sensor placed in the chamber (Servoflo, Lexington, MA) and then recorded. Oxygen was controlled with a relay valve for nitrogen gas inflow as previously reported (Schreier et al. 2013). Age‐matched Col1a1^+/+^ (*n* = 12) and Col1a1^R/R^ (*n* = 13) controls were placed in room air. The University of Wisconsin‐Madison Institutional Animal Care and Use Committee approved all procedures.

### Echocardiography

Immediately before RV catheterization, transthoracic echocardiography was done to assess cardiac function as previously described (Harris et al. 2002; Brody et al. 2012). Briefly, mice were anesthetized with isoflurane (1%) and maintained at 37°C via a heated platform. Mitral valve velocities in early and late diastole (MVE, MVA), ejection fraction (EF), and fractional shortening (FS) were determined from LV images acquired over at least three consecutive heartbeats. Aorta and pulmonary ejection times were evaluated.

### Hemodynamic measurements and ventricular function

Surgical preparation was based on established protocols (Tabima et al. 2010; Schreier et al. 2013; Golob et al. 2015). Anesthesia was induced with an intraperitoneal injection of urethane solution (1 mg/g body weight). Mice were then intubated and placed on a ventilator (Harvard Apparatus, Holliston, MA). A ventral midline skin incision was made from the lower mandible inferior to the xiphoid process as done previously (Tabima et al. 2010; Schreier et al. 2013). The thoracic cavity was entered through the sternum. The heart was exposed after retracting the chest wall (open chest procedure). Hydroxyethylstarch (6%, 2 mg/g body weight) was injected intravenously to restore vascular volumes as previously reported (Pacher et al. 2008; Porterfield et al. 2009). Systemic blood pressure was measured with a pressure catheter (Millar, Houston, TX) inserted from the common carotid artery and advanced to the aorta. Heart rate and systemic pressure were recorded and observed throughout the procedure. RV pressure‐volume loops were obtained using a catheterization procedure using a 1.2 F catheter inserted through the apex of the heart into the right ventricle as previously described (Tabima et al. 2010; Schreier et al. 2013; Golob et al. 2015). Catheter calibration was performed by measuring magnitude and phase in saline solutions of known conductivities. Commercial software (Notocord, Croissy Sur Seine, France) recorded RV pressure and volume waveforms simultaneously, and data were analyzed using a minimum of 10 consecutive cardiac cycles. RV function was quantified using established parameters including end‐systolic pressure (RVSP), maximum and minimum pressure derivatives (d*P*/d*t*_max_, d*P*/d*t*_min_), cardiac output (CO), effective arterial elastance (*E*_a_), and total pulmonary vascular resistance (TPVR) (Kelly et al. 1992; Schreier et al. 2013; Golob et al. 2015). To account for hypertension‐dependent weight changes, cardiac output was normalized by body weight (BW) to calculate the cardiac index (CI). RV contractility was assessed by end systolic elastance (*E*_es_), preload recruitable stroke work (PRSW), and the slope of d*P*/d*t*_max_‐end diastolic volume (*V*_ed_) relationship (Pacher et al. 2008) obtained from inferior vena cava occlusions (Tabima et al. 2010). Ventricular–vascular coupling efficiency was assessed using *E*_es_/*E*_a_ (Tabima et al. 2010). Cardiac energetics were assessed via pressure‐volume area (PVA), external mechanical work (EW), and ventricular mechanical efficiency (EW/PVA) as previously reported (Nozawa et al. 1988; Liu et al. 2014).

### Hematocrit and indices of RV hypertrophy

After RV catheterization, animals were euthanized, and blood samples were centrifuged to quantify hematocrit (Hct). RV hypertrophy was determined using the RV free wall weight normalized by the LV free wall weight plus the septum weight (S) (RV/[LV + S]; Fulton index) (Ciuclan et al. 2011; Wang et al. 2013b). RV free wall weight was also normalized to body weight (Borgdorff et al. 2013) and tibia length (TL) (Csiszar et al. 2009) to account for weight changes.

### Histology

Following catheterization and euthanasia, RVs were excised, fixed in 10% formalin, and preserved in 70% ethanol as previously reported by our group (Wang et al. 2013b). RV tissues were then embedded in paraffin, sectioned, and stained with picrosirius red to identify collagen. An inverted microscope (TE‐2000‐5; Nikon, Melville, NY) was used to acquire all images using a Spot CCD camera (Optical Analysis Systems, Nashua, NH). The area containing collagen was determined by color thresholding in a representative field of view by an observer blinded to the experimental condition using MetaVue software (Optical Analysis Systems). The collagen area was divided by the tissue area to calculate the collagen area percent. Using polarized light, the area positive for collagen subtypes was identified using a separate color thresholding scheme: areas of green and orange/yellow in the representative field of view were measured as an area percentage of the total image (Wang et al. 2013a). The percentages of type I and type III collagen were evaluated as the area percentages of green, and orange/yellow, respectively. Finally, perivascular fibrosis was quantified using the collagen area surrounding a vessel divided by the total vessel area (Nergui et al. 2014).

### Statistics

Data are reported as mean ± standard error. Parameters were evaluated using a two‐way analysis of variance (ANOVA) for strain (Col1a1^+/+^/Col1a1^R/R^) and treatment (control/HySu). Tukey's Honestly Significance Difference method was utilized to control the type I error when conducting multiple comparisons. Model assumptions for analyses were validated by examining normal probability plots. *E*_es_ was calculated as the slope of the linear fit to the end‐systolic pressure and volume points with a Pearson correlation coefficient of greater than 0.9. A two‐sided *P*‐value less than 0.05 was considered statistically significant. All analyses were conducted using R‐software version 3.2.2 (R Foundation for Statistical Computing, Vienna, Austria).

## Results

### RV hypertrophy and hematocrit changes

**Figure 1:**
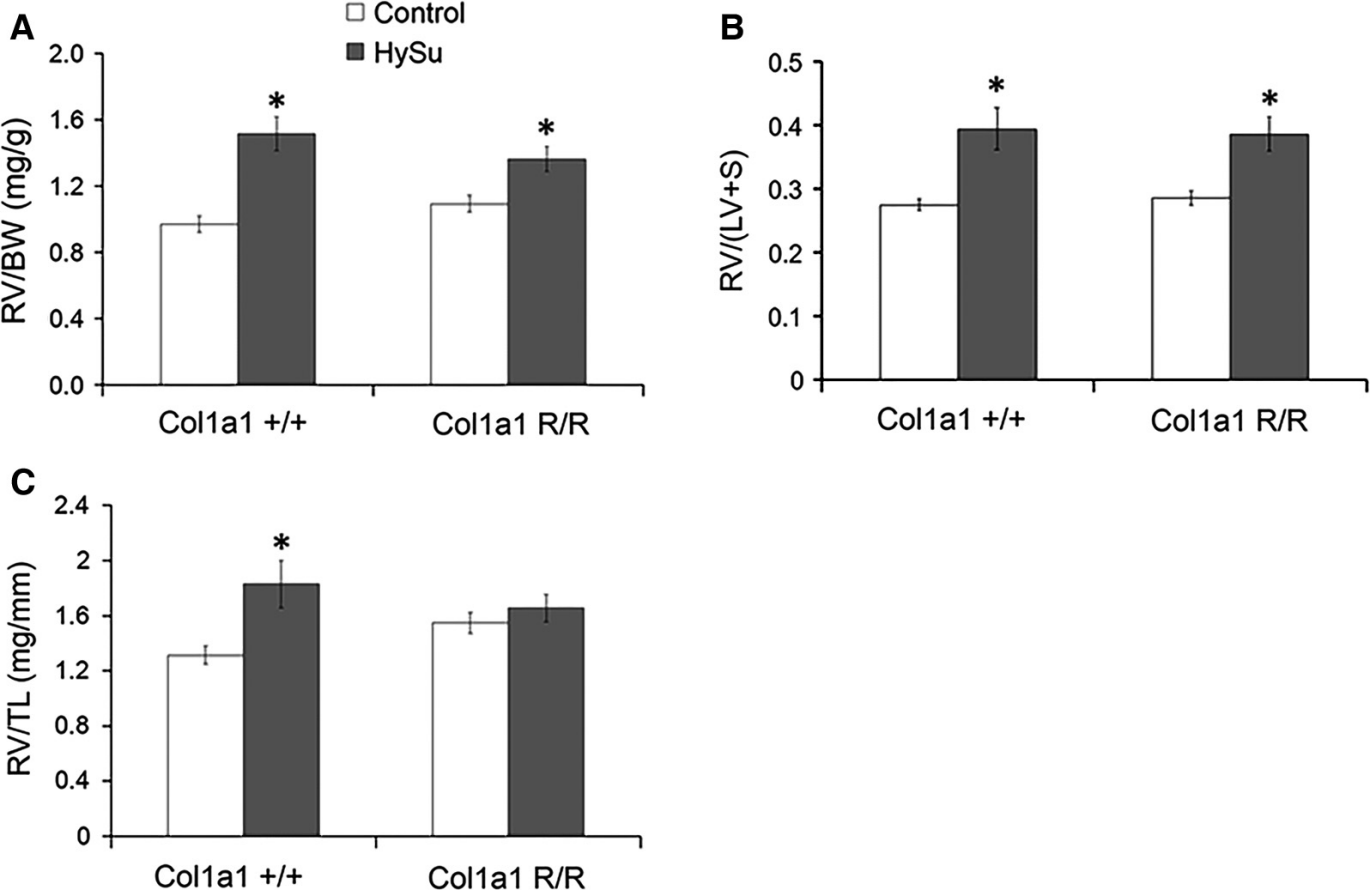
Right ventricle (RV) hypertrophy indices of control and HySu‐treated Col1a1 mice including (A) RV/BW ratio, (B) RV/(LV + S) (Fulton Index), and (C) RV/TL ratio. Both mouse strains had RV hypertrophy with HySu treatment evident from increased RV/BW and RV/(LV + S) ratios. Only wild‐type mice had RV hypertrophy as quantified by the RV/TL ratio. *P < 0.05 versus control of the same strain.

Differences in RV hypertrophy indices and hematocrit were evaluated between control and HySu‐treated mice. All HySu‐treated mice exhibited RV hypertrophy as evident by larger RV/BW and RV/(LV + S) ratios (Fig. 1A,B), and the extent was greater in Col1a1^+/+^ mice. Only HySu‐treated Col1a1^+/+^ mice demonstrated RV hypertrophy based on the RV/TL ratio (Fig. 1C; *P* = 0.027). Body weight and LV + S weight were lower in mice exposed to HySu treatment compared to controls, but TL was not affected by the collagen mutation or treatment (Table 1). These data demonstrate that isolated RV hypertrophy is more pronounced in Col1a1^+/+^ mice. As expected, hematocrit was increased with HySu exposure in both genotypes (Table 1). No differences were evident between wild‐type and mutant mice in the absence of HySu treatment.

**Table 1:** Reference parameters for assessment of RV hypertrophy and hematocrit in control and HySu‐treated Col1a1 mice

Strain treatment group	Col1a1^+/+^ control	Col1a1^+/+^ HySu	Col1a1^R/R^ control	Col1a1^R/R^HySu
RV (g)	22 ± 1	29 ± 3	25 ± 1	27 ± 2
BW (g)	23 ± 1	19 ± 1^a^	24 ± 1	20 ± 1^a^
LV + S (mg)	81 ± 4	71 ± 3	87 ± 3	70 ± 4^a^
TL (mm)	17 ± 0	16 ± 1	16 ± 1	16 ± 0
Hct (%)	46 ± 1	66 ± 4^a^	45 ± 2	61 ± 2^a^

Values are mean ± standard error. RV, right ventricle; BW, body weight; Hct, hematocrit; LV, left ventricle; S, septum; TL, tibia length.

a *P* < 0.05 versus control of the same strain.

### Hemodynamics and RV function changes

**Figure 2:**
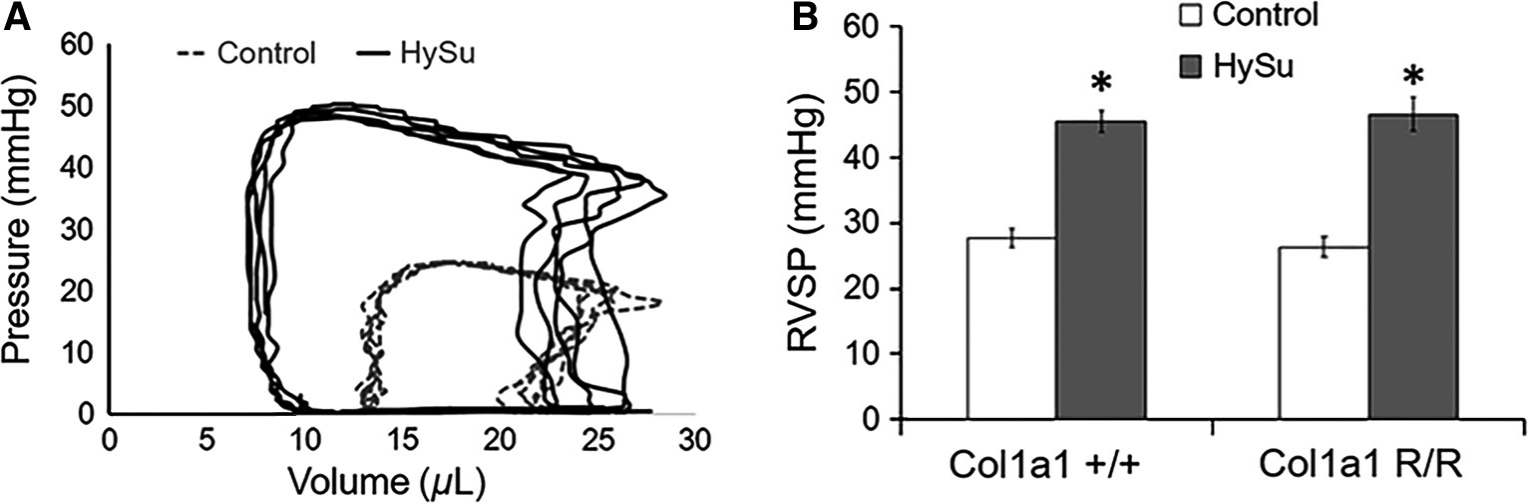
Right ventricular systolic pressure (RVSP) is illustrated using (A) representative pressure‐volume loops from mutant mice and (B) comparisons between controls and HySu‐treated Col1a1 mice. RVSP was increased with HySu‐treatment in both strains. *P < 0.05 versus control of the same strain.

Representative pressure‐volume loops for control and HySu groups are illustrated in Figure 2A. RVSP was elevated with HySu treatment (Fig. 2A,B), which confirms the development of PAH, and the severity was comparable in the two genotypes. RV d*P*/d*t*_max_ and d*P*/d*t*_min_ were higher and lower, respectively, with HySu treatment (Table 2). Commonly used LV functional metrics, including EF and FS were not altered, indicating the HySu treatment did not affect the LV as measured by echocardiography (Table 2).

**Table 2:** Hemodynamic and ventricular function parameters in control and HySu‐treated Col1a1 mice

Strain treatment group	Col1a1^+/+^ control	Col1a1^+/+^ HySu	Col1a1^R/R^ control	Col1a1^R/R^ HySu
*Right ventricle/pulmonary circulation*				
d*P*/d*t*_max_ (mmHg/s)	2249 ± 129	3559 ± 346^a^	2096 ± 135	3736 ± 267^a^
d*P*/d*t*_min_ (mmHg/s)	−1752 ± 94	−2944 ± 221^a^	−1672 ± 123	−3099 ± 190^a^
CI (*μ*L/min g)	330 ± 34	313 ± 41	347 ± 35	419 ± 44
PA ET (ms)	54.5 ± 2.2	51.5 ± 1.7	57.6 ± 1.0	47.8 ± 6.0
*Left ventricle/systemic circulation*				
EF (%)	69 ± 3	73 ± 4	74 ± 2	67 ± 2
FS (%)	39 ± 2	43 ± 3	43 ± 2	37 ± 2
SP (mmHg)	74 ± 10	78 ± 14	67 ± 13	77 ± 9
MVE (mm/s)	785 ± 41	693 ± 31	695 ± 29	745 ± 65
MVA (mm/s)	580 ± 40	471 ± 17	557 ± 25	473 ± 48
AO ET (ms)	47.7 ± 1.6	47.5 ± 2.0	51.1 ± 1.1	47.6 ± 1.0

Values are mean ± standard error. d*P*/d*t*_max_ and d*P*/d*t*_min_, maximal and minimal derivatives of pressure; CI, cardiac index; PA, pulmonary artery; ET, ejection time; EF, ejection fraction; FS, fractional shortening; SP, systolic pressure; MVE, mitral valve velocity in early diastole; MVA, mitral valve velocity in late diastole; AO, aorta.

a *P* < 0.05 versus control of the same strain.

**Figure 3:**
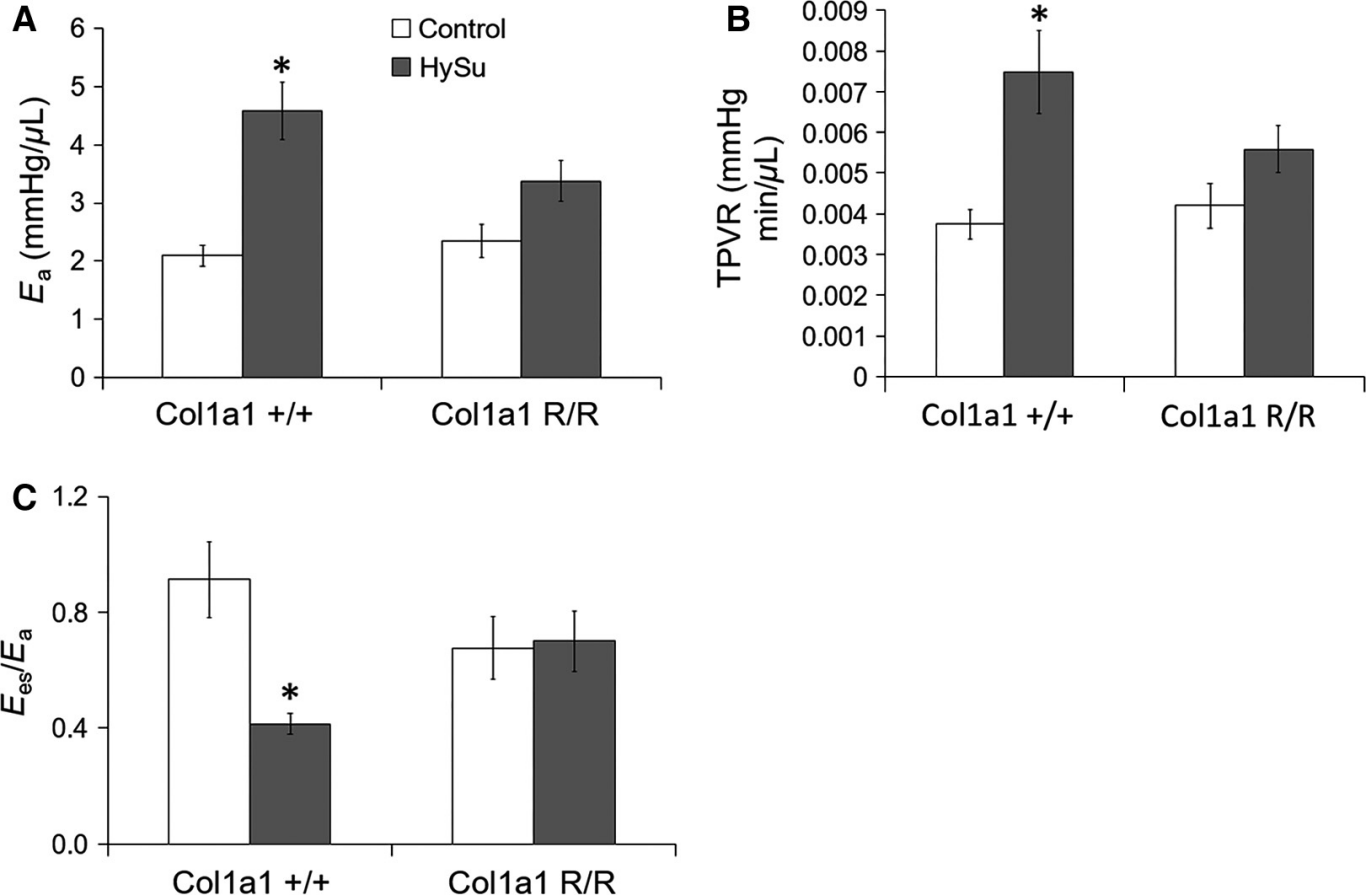
Afterload and ventricular–vascular coupling efficiency metrics of control and HySu‐treated Col1a1 mice including (A) arterial elastance, (B) total pulmonary vascular resistance, and (C) ventricular–vascular coupling efficiency. Afterload increased with HySu treatment in both strains, and the increase was more prominent in the wild‐type mice. Ventricular–vascular coupling was decreased in wild‐type mice with HySu treatment. *P < 0.05 versus control of the same strain.

**Figure 4:**
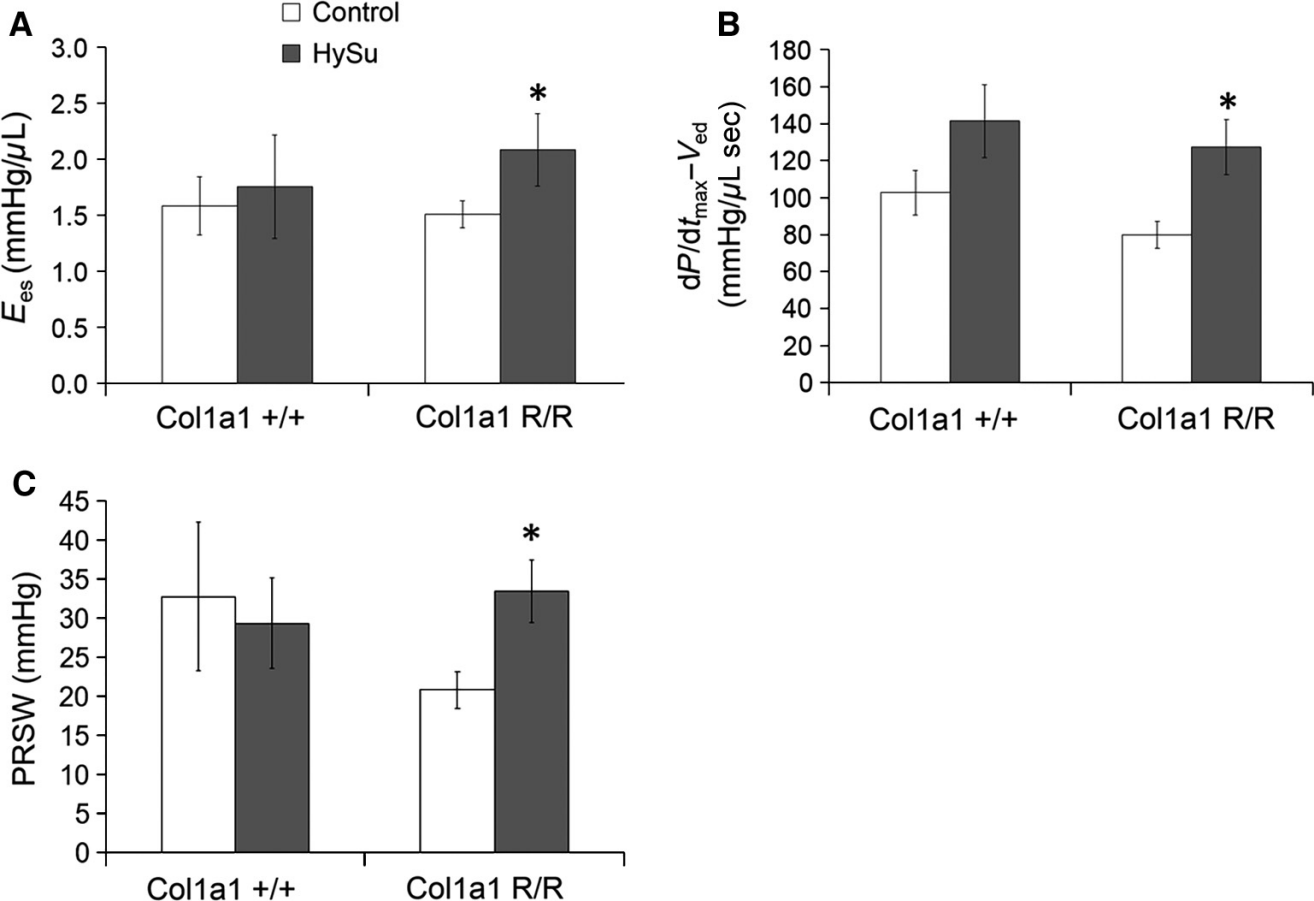
Contractility measures of control and HySu‐treated Col1a1 mice including (A) end‐systolic elastance, (B) maximum pressure derivative‐end diastolic volume relationship, and (C) preload recruitable stroke work. With HySu treatment, contractility was increased in the mutant mice as shown by increases in *E*_es_, d*P*/d*t*_max_‐*V*_ed_, and PRSW. *P < 0.05 versus control of the same strain.

Right ventricular contractility and afterload were assessed to determine the cardiovascular effects of impaired collagen turnover in PAH. Interestingly, despite equivalent RVSP, *E*_a_ and TPVR increased significantly only in the Col1a1^+/+^ mice (Fig. 3A,B; *E*_a_, *P *= 10^−5^; TPVR, *P* = 0.002), and RV contractility quantified by *E*_es_, d*P*/d*t*_max_‐*V*_ed_, and PRSW was larger only in the Col1a1^R/R^ mice (Fig. 4; Ees, *P* = 0.039, d*P*/d*t*_max_‐*V*_ed_, *P* = 0.037; PRSW, *P* = 0.021). The combined increase in RV afterload and a lack of increase in RV contractility led to a decrease in the ventricular–vascular coupling efficiency *E*_es_/*E*_a_ in the Col1a1^+/+^ mice, indicating RV dysfunction (Fig. 3C; *P* = 0.011). In contrast, RV afterload (*E*_a_) in the Col1a1^R/R^ strain did not change while RV contractility increased such that *E*_es_/*E*_a_ was maintained, demonstrating preserved RV function in the Col1a1^R/R^ strain. These results indicate that impaired collagen turnover limited RV overload and prevented ventricular–vascular uncoupling in severe PAH.

### Cardiac energetics changes

**Figure 5:**
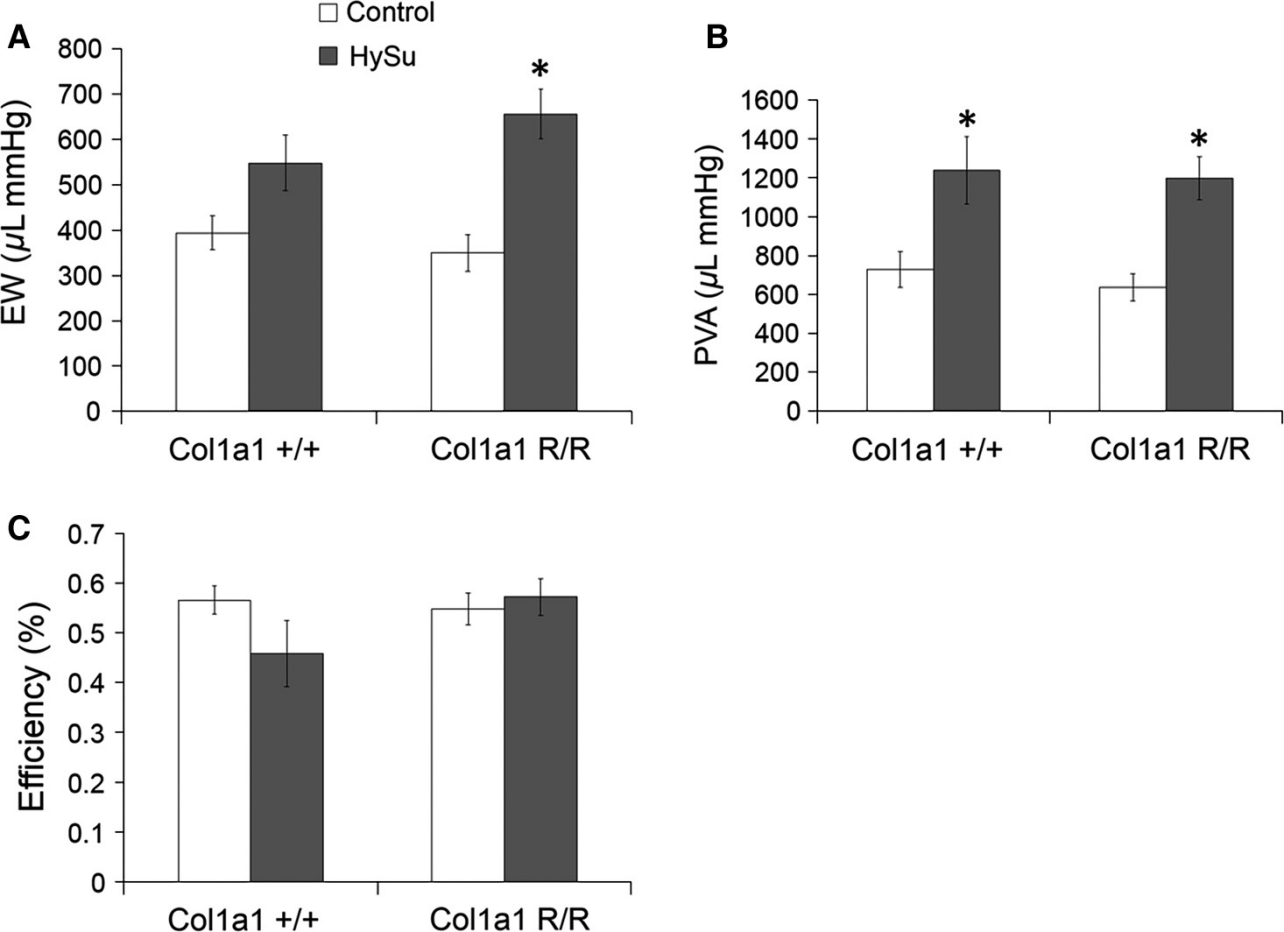
Cardiac energetics of control and HySu‐treated Col1a1 mice including (A) external mechanical work, (B) pressure‐volume area, and (C) ventricular mechanical efficiency. External mechanical work and pressure‐volume area were increased with HySu treatment in mutant mice and in both strains, respectively. Cardiac mechanical efficiency was not significantly altered in either strain with HySu treatment. *P < 0.05 versus control of the same strain.

Since myocardial oxygen demand becomes greater with increased afterload (Wang and Chesler 2011), we examined the cardiac energetic changes with HySu‐treatment. Ventricular energy output as quantified by EW increased significantly in the Col1a1^R/R^ mice (Fig. 5A; *P *= 10^−4^). The total mechanical energy from ventricular contraction (PVA) increased to a similar degree with HySu treatment in both strains (Fig. 5B), and ventricular mechanical efficiency (EW/PVA) was maintained with HySu treatment in both strains (Fig. 5C).

### Collagen accumulation

**Figure 6:**
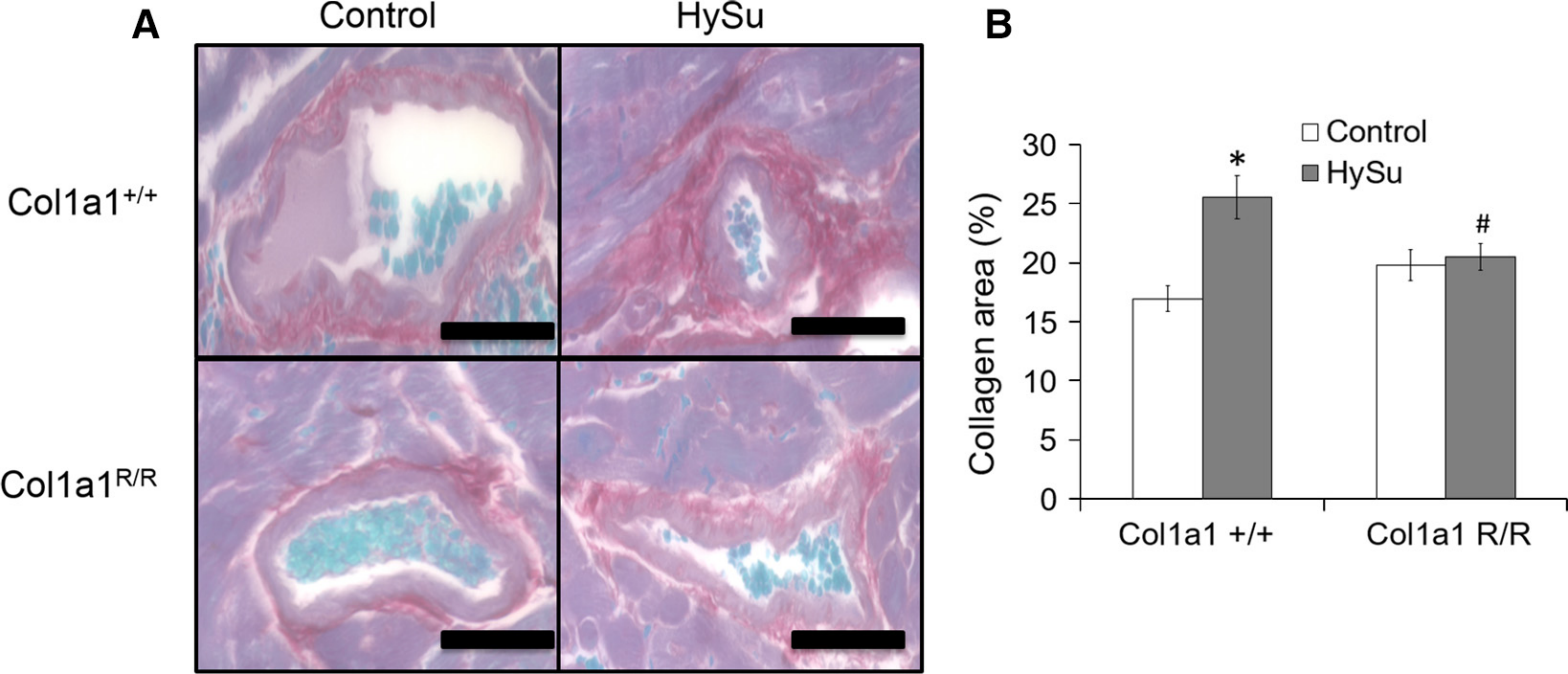
Right ventricle collagen content in control and HySu‐treated Col1a1 mice from (A) representative picrosirius red staining of perivascular vessels in all groups (scale bar = 100 *μ*m) and (B) total collagen area percent (collagen area/vessel wall area). Collagen accumulation is evident in the Col1a1^+/+^ HySu group but absent in the Col1a1^R/R^ group. *P < 0.05 versus control of the same strain. #*P* < 0.05 versus Col1a1^+/+^ HySu.

Representative histological images of perivascular fibrosis are illustrated with picrosirius red staining for collagen (Fig. 6A). Total collagen content expressed as a tissue area percentage from color thresholding in the RV was increased with HySu treatment in Col1a1^+/+^ mice, and the increase was negligible in the Col1a1^R/R^ mice (Fig. 6B). Collagen subtypes were then examined using polarized light imaging of the same tissue. Similar to total collagen content percentage, collagen subtype percentage results are presented as a percentage of tissue area. The ratio of collagen type I to type III was increased in HySu‐treated Col1a1^+/+^ mice (*P* = 0.004) and did not change in HySu‐treated Col1a1^R/R^ mice (Table 3).

**Table 3:** Right ventricle collagen content in control and HySu‐treated Col1a1 mice

Strain treatment group	Col1a1^+/+^ control	Col1a1^+/+^ HySu	Col1a1^R/R^ control	Col1a1^R/R^ HySu
Type I (%)	9.0 ± 0.9	12.7 ± 1.3	12.2 ± 2.3	10.9 ± 1.1
Type III (%)	2.9 ± 0.3	2.0 ± 0.3	2.8 ± 0.7	3.2 ± 0.5
Type I/Type III	3.0 ± 0.24	8.8 ± 1.8^a^	4.3 ± 1.2	3.4 ± 0.8^b^

Values are mean ± standard error.

a *P* < 0.05 versus control of the same strain.

b *P* < 0.05 versus Col1a1^+/+^ HySu.

## Discussion

**Figure 7:**
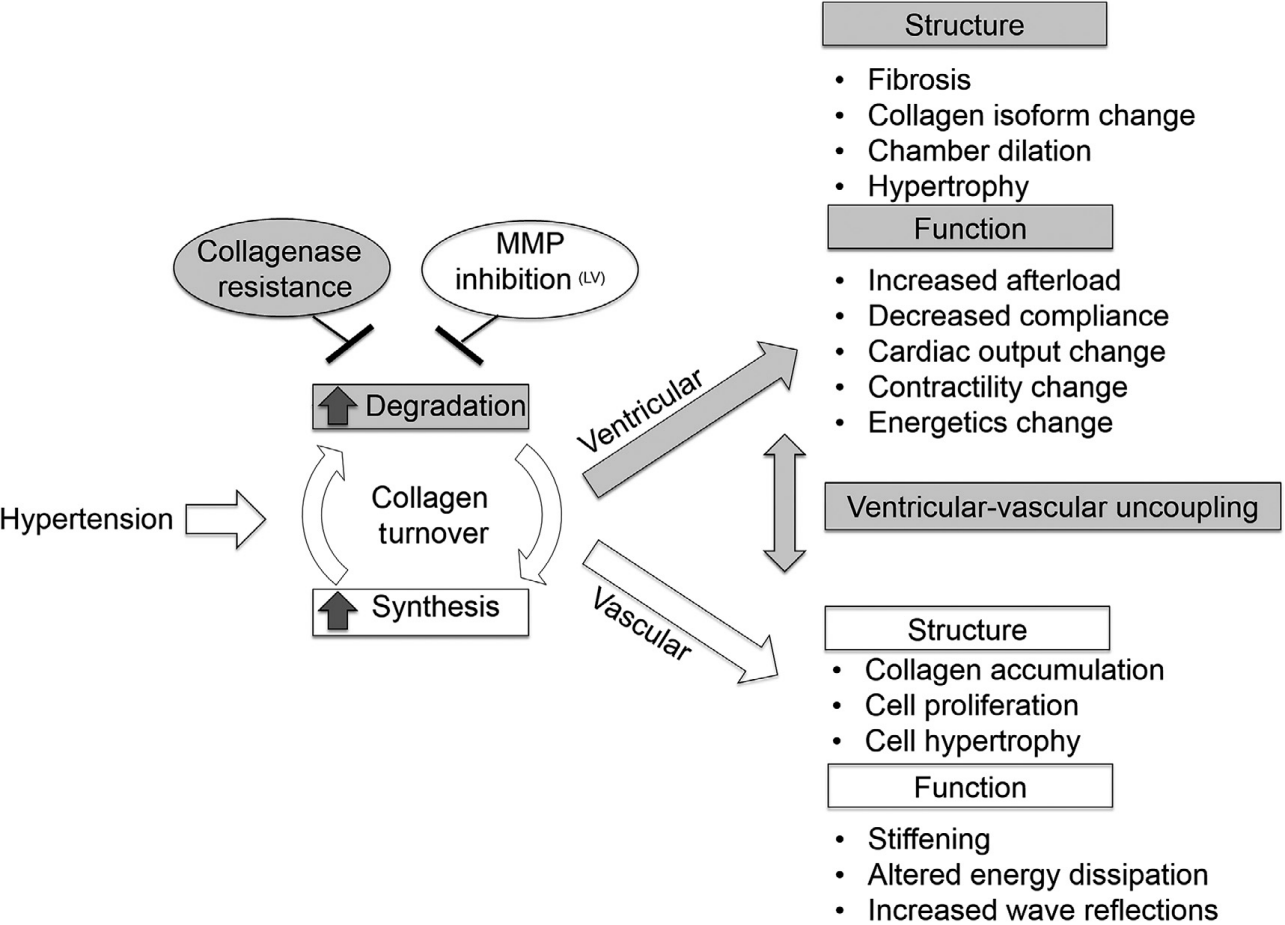
The findings and mechanistic approach to studying the impact of collagen turnover on right ventricular (RV) structure and function in response to pulmonary hypertension. We investigated the RV response to limiting collagen turnover via impaired degradation using mice with type I collagen resistant to degradation. Collagen turnover is defined as the balance of collagen degradation and synthesis. Other studies have examined the effect of limiting collagen turnover through MMP inhibition or limiting collagen synthesis (Ducharme et al. 2000; Peterson et al. 2001; Schreier et al. 2013).

The major findings of this study are that limiting collagen turnover through impaired collagen degradation using the Col1a1 mouse model prevents RV hypertrophy, fibrosis, and collagen type I/III ratio changes with chronic pressure overload, which subsequently attenuated RV dysfunction. These results indicate a protective role of down‐regulating collagen turnover in RV adaptation to pressure overload. While impaired collagen degradation did not protect against the development of severe PAH (RVSP ~46 mmHg for both genotypes), it did limit the increase in steady RV afterload (TPVR), which likely contributed to attenuated RV dysfunction and maintained ventricular–vascular coupling. A summary schematic of our approach and findings (shaded boxes) regarding the impact of collagen turnover on RV structure‐function changes in response to pulmonary hypertension is provided in Figure 7.

Right ventricular hypertrophy was evident in all HySu‐treated mice by larger RV/BW and RV/(LV + S) (Fulton index) and in HySu‐treated Col1a1^+/+^ mice by larger RV/TL (Fig. 1). RV/BW and Fulton index are the most common metrics used to assess RV hypertrophy, and increases in these metrics in preclinical models of PAH are nearly universally reported (Ciuclan et al. 2011; Wang et al. 2013c; Liu et al. 2014, 2015; Nergui et al. 2014; Mendes‐Ferreira et al. 2015). However, RV/BW and Fulton index can be confounded by body weight or LV + S weight changes, respectively. TL is a more robust reference parameter when assessing cardiac hypertrophy (Yin et al. 1982). The RV/TL ratio was significantly larger in HySu‐treated Col1a1^+/+^ mice and this was associated with increased collagen deposition. In contrast, the RV/TL ratio was maintained in HySu‐treated Col1a1^R/R^ mice and associated with negligible collagen accumulation. These results confirm RV hypertrophy in HySu‐treated Col1a1^+/+^ mice associated with increased collagen deposition and indicate that RV hypertrophy was limited in HySu‐treated Col1a1^R/R^ mice.

Right ventricular fibrosis was evident in the HySu‐treated Col1a1^+/+^ mice, accompanied by a pronounced increase in the collagen type I/type III ratio. RV fibrosis is generally understood to impair diastolic function, but its effect on systolic function is less clear and may depend on the stage of the disease (Brower et al. 2006). Changes in collagen subtype ratios have been previously observed in LV remodeling in response to experimentally induced or genetic hypertension (Mukherjee and Sen 1990, 1993; Burgess et al. 1996), biventricular experimental myocardial infarction (Wei et al. 1999), and in patients with dilated myopathy (Marijianowski et al. 1995; Pauschinger et al. 1999). Collagen type I is characterized by thicker fibers that provide strength and stiffness, whereas collagen type III is characterized by thinner fibers that are more compliant and form a thin elastic network (Lapiere et al. 1977). We speculate that increased collagen type I may reinforce the thick collagen fiber network to accommodate the increased loading on the RV in PAH, which is supported by the collagen type I‐dominated fibrosis seen in the pressure overloaded rat LV (Jalil et al. 1988). An increase in collagen type III may result in a higher degree of compliance as seen previously in the LV from rodent models (Borg et al. 1981) or may increase collagen fiber density around RV myocytes in early stages of cardiac hypertrophy due to pressure‐overload (Honda et al. 1992). It is well known that collagen type I contributes to tendon (Lapiere et al. 1977) as well as pulmonary artery mechanics (Wang et al. 2013a), and the collagen type I/III ratio shift is well documented in preclinical and clinical studies. However, the differential contribution of each collagen subtype to RV mechanics and function in PAH is unknown and warrants future investigation. In addition to increased content and altered composition, high collagen turnover may result in misalignment and gaps in the supporting collagen structure surrounding cardiomyocytes, resulting in reduced force development and shortening (Baicu et al. 2003). Our results indicate that reducing collagen turnover by inhibiting degradation attenuates the RV structural remodeling in PAH.

While the RVSP after HySu treatment was equivalent in both strains and high enough to indicate severe PAH, RV afterload assessed by effective arterial elastance and total pulmonary vascular resistance was not in the mutant mice. This difference can be attributed to different effects of collagenase‐resistance on the steady and pulsatile components of RV afterload. The steady component of RV afterload is largely determined by narrowing of small distal arteries and can be quantified by PVR or approximated by TPVR. The pulsatile component of the RV afterload is determined by stiffening of proximal and distal arteries and can be quantified by hemodynamic metrics affected by pulmonary vascular stiffness such as pulse wave velocity or characteristic impedance (Wang and Chesler 2011). We did not measure the pulsatile RV afterload here, which would require simultaneous pulmonary arterial pressure and flow measurements (Tabima et al. 2012), but the equivalent RVSP suggests a higher pulsatile RV afterload in the mutant mice compared to the wild‐type mice. We have previously observed maintained or slightly reduced (Ooi et al. 2010; Tabima et al. 2012; Wang and Chesler 2012; Wang et al. 2013a) pulmonary vascular stiffness in Col1a1^R/R^ mice compared to Col1a1^+/+^ mice exposed to chronic hypoxia but in all of these experiments the generated PAH was mild. Therefore, the relative impacts of impaired collagen turnover on RV afterload changes in response to severe PAH are as yet unclear.

The effective arterial elastance, *E*_a_, while having the benefit of being a single number that captures ventricular afterload, is an as‐yet ill‐defined combination of steady and pulsatile afterload. In the systemic circulation, *E*_a_ is nearly three times more sensitive to resistance than compliance in both healthy and hypertensive subjects (Chemla et al. 2003). However, the sensitivity of LV *E*_a_ to resistance and compliance has been shown to vary with exercise (Otsuki et al. 2006). The relative contributions of resistance and compliance to RV *E*_a_ in healthy and disease states, such as chronic pressure overload, are unknown.

Differences in effective arterial elastance between the strains contributed to the difference in ventricular–vascular coupling efficiencies with HySu treatment. In mild PAH, an increase in RV afterload (*E*_a_) has been shown to cause an increase in RV contractility (*E*_es_) such that ventricular–vascular coupling (*E*_es_/*E*_a_) is maintained (Wang et al. 2013c). As disease progresses, RV contractility cannot match the extent of RV afterload increase, which results in ventricular–vascular uncoupling (Tabima et al. 2010; Sanz et al. 2012; Aguero et al. 2014; Borgdorff et al. 2015; Guihaire et al. 2015). In this study, *E*_es_ did not increase in Col1a1^+/+^ mice, and the increase in *E*_a_ without a concurrent increase in *E*_es_ led to decreased ventricular–vascular coupling indicative of RV dysfunction. In contrast, in HySu‐treated Col1a1^R/R^ mice, although the increase in RV afterload was mild (and insignificant), RV contractility increased to match it such that ventricular–vascular coupling was unchanged.

Increased RV afterload leads to a larger demand for myocardial oxygen, which was assessed using cardiac energetic parameters. PVA, which is correlated with myocardial oxygen consumption, was increased to a similar extent in all HySu‐treated mice. While the total oxygen demand increase was similar between the two strains, the EW increase was significant only in mutant mice. In a canine model, LV EW/PVA was shown to increase with *E*_es_ and decrease with *E*_a_ (Nozawa et al. 1988). If true in the mouse RV, the unchanged *E*_es_ and elevated *E*_a_ in HySu‐treated wild‐type mice should decrease the EW/PVA, which is consistent with our observations. Similarly, the increase in *E*_es_ and attenuated increase in *E*_a_ observed in HySu‐treated mutant mice should increase the mechanical efficiency, which is also consistent with our results although the changes were not significant. Our results suggest that (1) RV EW/PVA shows similar trends due to *E*_es_ and *E*_a_ compared to the previously reported LV EW/PVA and (2) reducing collagen turnover though collagenase‐resistance in the Col1a1 protects against impaired ventricular mechanical efficiency.

### Limitations

A more comprehensive and RV function‐independent measurement of the RV afterload can be done by measuring pulmonary vascular impedance ex vivo (Vanderpool and Chesler 2011). Given the sensitivity of RV remodeling to the steady and pulsatile components of RV afterload, future work must focus on quantifying the impact of each of these components separately on RV functional adaptation. In the mouse model used, the collagen type I defect affects the entire cardiovascular system, and it is difficult to distinguish its effect on the RV alone. It will be important that future work distinguish between cardiac and vascular effects (Lindsey et al. 2003; Mendes‐Ferreira et al. 2015) of collagen turnover, perhaps with cardiac‐specific collagen turnover impairment. Since the trends were similar in male and female mice, we combined our results in this study to isolate the effect of the collagen mutation. It is possible that interactions between sex and treatment exist, which is an important future research direction.

In prior studies, we have observed significant changes in pulmonary vascular and RV function with 10 or 15 days of chronic hypoxia or 14 days of chronic hypoxia plus SUGEN treatment (Kobs et al. 2005; Ooi et al. 2010; Tabima and Chesler 2010; Wang et al. 2013c). The time frame required to observe changes in RV function in response to PAH is likely longer than that required to observe pulmonary vascular changes; previously we have observed changes in RV function with 14, 21, and 28 days of HySu treatment (Wang et al. 2013c). Here, we chose a relatively short 14 day exposure to permit comparison to prior results (Wang et al. 2013c). Our results indicate this treatment duration was sufficient to induce significant changes in RV function in wild‐type mice. Finally, preventing collagen degradation may affect collagen synthesis and MMP/tissue inhibitors of MMPs (TIMP) activity, so measurements of MMP/TIMP and mRNA expression warrant future study.

In summary, our results show that limiting collagen turnover via inhibiting collagen degradation through use of the Col1a1 mouse attenuates the maladaptive changes in right ventricular‐pulmonary vascular coupling and prevents RV hypertrophy and fibrosis in PAH. Understanding the role of collagen turnover in RV hypertrophy and function may lead to targeted clinical treatments to prevent maladaptive RV structural and functional changes in PAH.

## Conflicts of Interest

No conflicts of interest, financial or otherwise, are declared by the authors.

## References

[phy212815-bib-0001] Aguero, J. , K. Ishikawa , L. Hadri , C. Santos‐Gallego , K. Fish , N. Hammoudi , et al. 2014 Characterization of right ventricular remodeling and failure in a chronic pulmonary hypertension model. Am. J. Physiol. Heart Circ. Physiol. 307:H1204–H1215. 25158063 10.1152/ajpheart.00246.2014 PMC4200337

[phy212815-bib-0002] Baicu, C. F. , J. D. Stroud , V. A. Livesay , E. Hapke , J. Holder , F. G. Spinale , et al. 2003 Changes in extracellular collagen matrix alter myocardial systolic performance. Am. J. Physiol. Heart Circ. Physiol. 284:H122–H132. 12485818 10.1152/ajpheart.00233.2002

[phy212815-bib-0003] Borg, T. K. , W. F. Ranson , F. A. Moslehy , and J. B. Caulfield . 1981 Structural basis of ventricular stiffness. Lab. Invest. 44:49–54. 7453130

[phy212815-bib-0004] Borgdorff, M. A. , B. Bartelds , M. G. Dickinson , P. Steendijk , M. de Vroomen , and R. M. Berger . 2013 Distinct loading conditions reveal various patterns of right ventricular adaptation. Am. J. Physiol. Heart Circ. Physiol. 305:H354–H364. 23729212 10.1152/ajpheart.00180.2013

[phy212815-bib-0005] Borgdorff, M. A. , M. G. Dickinson , R. M. Berger , and B. Bartelds . 2015 Right ventricular failure due to chronic pressure load: what have we learned in animal models since the NIH working group statement? Heart Fail. Rev. 20:475–491. 25771982 10.1007/s10741-015-9479-6 PMC4463984

[phy212815-bib-0006] Brody, M. J. , T. A. Hacker , J. R. Patel , L. Feng , J. Sadoshima , S. G. Tevosian , et al. 2012 Ablation of the cardiac‐specific gene leucine‐rich repeat containing 10 (Lrrc10) results in dilated cardiomyopathy. PLoS ONE 7:e51621. 23236519 10.1371/journal.pone.0051621 PMC3517560

[phy212815-bib-0007] Brower, G. L. , J. D. Gardner , M. F. Forman , D. B. Murray , T. Voloshenyuk , S. P. Levick , et al. 2006 The relationship between myocardial extracellular matrix remodeling and ventricular function. Eur. J. Cardiothorac. Surg. 30:604–610. 16935520 10.1016/j.ejcts.2006.07.006

[phy212815-bib-0008] Burgess, M. L. , J. Buggy , R. L. Price , F. L. Abel , L. Terracio , A. M. Samarel , et al. 1996 Exercise‐ and hypertension‐induced collagen changes are related to left ventricular function in rat hearts. Am. J. Physiol. 270:H151–H159. 8769746 10.1152/ajpheart.1996.270.1.H151

[phy212815-bib-0009] Chemla, D. , I. Antony , Y. Lecarpentier , and A. Nitenberg . 2003 Contribution of systemic vascular resistance and total arterial compliance to effective arterial elastance in humans. Am. J. Physiol. Heart Circ. Physiol. 285:H614–H620. 12689857 10.1152/ajpheart.00823.2002

[phy212815-bib-0010] Ciuclan, L. , O. Bonneau , M. Hussey , N. Duggan , A. M. Holmes , R. Good , et al. 2011 A novel murine model of severe pulmonary arterial hypertension. Am. J. Respir. Crit. Care Med. 184:1171–1182. 21868504 10.1164/rccm.201103-0412OC

[phy212815-bib-0011] Csiszar, A. , N. Labinskyy , S. Olson , J. T. Pinto , S. Gupte , J. M. Wu , et al. 2009 Resveratrol prevents monocrotaline‐induced pulmonary hypertension in rats. Hypertension 54:668–675. 19597040 10.1161/HYPERTENSIONAHA.109.133397 PMC2745434

[phy212815-bib-0012] D'Armiento, J. 2002 Matrix metalloproteinase disruption of the extracellular matrix and cardiac dysfunction. Trends Cardiovasc. Med. 12:97–101. 12007733 10.1016/s1050-1738(01)00160-8

[phy212815-bib-0013] Ducharme, A. , S. Frantz , M. Aikawa , E. Rabkin , M. Lindsey , L. E. Rohde , et al. 2000 Targeted deletion of matrix metalloproteinase‐9 attenuates left ventricular enlargement and collagen accumulation after experimental myocardial infarction. J. Clin. Invest. 106:55–62. 10880048 10.1172/JCI8768 PMC517910

[phy212815-bib-0014] Golob, M. J. , L. Tian , Z. Wang , T. A. Zimmerman , C. A. Caneba , T. A. Hacker , et al. 2015 Mitochondria DNA mutations cause sex‐dependent development of hypertension and alterations in cardiovascular function. J. Biomech. 48:405–412. 25582357 10.1016/j.jbiomech.2014.12.044 PMC4306604

[phy212815-bib-0015] Guihaire, J. , P. E. Noly , S. Schrepfer , and O. Mercier . 2015 Advancing knowledge of right ventricular pathophysiology in chronic pressure overload: insights from experimental studies. Arch. Cardiovasc. Dis. 108:519–529. 26184869 10.1016/j.acvd.2015.05.008

[phy212815-bib-0016] Harris, S. P. , C. R. Bartley , T. A. Hacker , K. S. McDonald , P. S. Douglas , M. L. Greaser , et al. 2002 Hypertrophic cardiomyopathy in cardiac myosin binding protein‐C knockout mice. Circ. Res. 90:594–601. 11909824 10.1161/01.res.0000012222.70819.64

[phy212815-bib-0017] Honda, M. , S. Yamada , Y. Goto , S. Ishikawa , H. Yoshikane , Y. Ishinaga , et al. 1992 Biochemical and structural remodeling of collagen in the right ventricular hypertrophy induced by monocrotaline. Jpn. Circ. J. 56:392–403. 1533690 10.1253/jcj.56.392

[phy212815-bib-0018] Jalil, J. E. , C. W. Doering , J. S. Janicki , R. Pick , W. A. Clark , C. Abrahams , et al. 1988 Structural vs. contractile protein remodeling and myocardial stiffness in hypertrophied rat left ventricle. J. Mol. Cell. Cardiol. 20:1179–1187. 2470910 10.1016/0022-2828(88)90597-4

[phy212815-bib-0019] Kelly, R. P. , C. T. Ting , T. M. Yang , C. P. Liu , W. L. Maughan , M. S. Chang , et al. 1992 Effective arterial elastance as index of arterial vascular load in humans. Circulation 86:513–521. 1638719 10.1161/01.cir.86.2.513

[phy212815-bib-0020] Kobs, R. W. , N. E. Muvarak , J. C. Eickhoff , and N. C. Chesler . 2005 Linked mechanical and biological aspects of remodeling in mouse pulmonary arteries with hypoxia‐induced hypertension. Am. J. Physiol. Heart Circ. Physiol. 288:H1209–H1217. 15528223 10.1152/ajpheart.01129.2003

[phy212815-bib-0021] Lapiere, C. M. , B. Nusgens , and G. Pierard . 1977 Interaction between collagen type I and type III in conditioning bundles organization. Connect. Tissue Res. 5:21–29. 141359 10.3109/03008207709152608

[phy212815-bib-0022] Lindsey, M. L. , J. Yoshioka , C. MacGillivray , S. Muangman , J. Gannon , A. Verghese , et al. 2003 Effect of a cleavage‐resistant collagen mutation on left ventricular remodeling. Circ. Res. 93:238–245. 12855673 10.1161/01.RES.0000085580.45279.60

[phy212815-bib-0023] Liu, X. , H. Wu , M. Byrne , J. Jeffrey , S. Krane , and R. Jaenisch . 1995 A targeted mutation at the known collagenase cleavage site in mouse type I collagen impairs tissue remodeling. J. Cell Biol. 130:227–237. 7790374 10.1083/jcb.130.1.227 PMC2120510

[phy212815-bib-0024] Liu, A. , D. Schreier , L. Tian , J. C. Eickhoff , Z. Wang , T. A. Hacker , et al. 2014 Direct and indirect protection of right ventricular function by estrogen in an experimental model of pulmonary arterial hypertension. Am. J. Physiol. Heart Circ. Physiol. 307:H273–H283. 24906919 10.1152/ajpheart.00758.2013 PMC4121651

[phy212815-bib-0025] Liu, A. , L. Tian , M. Golob , J. C. Eickhoff , M. Boston , and N. C. Chesler . 2015 17*β*‐estradiol attenuates conduit pulmonary artery mechanical property changes with pulmonary arterial hypertension. Hypertension 66:1082–1088. 26418020 10.1161/HYPERTENSIONAHA.115.05843 PMC4600044

[phy212815-bib-0026] Marijianowski, M. M. , P. Teeling , J. Mann , and A. E. Becker . 1995 Dilated cardiomyopathy is associated with an increase in the type I/type III collagen ratio: a quantitative assessment. J. Am. Coll. Cardiol. 25:1263–1272. 7722119 10.1016/0735-1097(94)00557-7

[phy212815-bib-0027] McLaughlin, V. V. , S. L. Archer , D. B. Badesch , R. J. Barst , H. W. Farber , J. R. Lindner , et al.; Accf/Aha . 2009 ACCF/AHA 2009 expert consensus document on pulmonary hypertension: a report of the American College of Cardiology Foundation Task Force on Expert Consensus Documents and the American Heart Association: developed in collaboration with the American College of Chest Physicians, American Thoracic Society, Inc., and the Pulmonary Hypertension Association. Circulation 119:2250–2294. 19332472 10.1161/CIRCULATIONAHA.109.192230

[phy212815-bib-0028] Mendes‐Ferreira, P. , C. Maia‐Rocha , R. Adao , M. J. Mendes , D. Santos‐Ribeiro , B. S. Alves , et al. 2015 Neuregulin‐1 improves right ventricular function and attenuates experimental pulmonary arterial hypertension. Cardiovasc. Res. 1:44–54. 26503987 10.1093/cvr/cvv244

[phy212815-bib-0029] Mukherjee, D. , and S. Sen . 1990 Collagen phenotypes during development and regression of myocardial hypertrophy in spontaneously hypertensive rats. Circ. Res. 67:1474–1480. 2147130 10.1161/01.res.67.6.1474

[phy212815-bib-0030] Mukherjee, D. , and S. Sen . 1993 Alteration of cardiac collagen phenotypes in hypertensive hypertrophy: role of blood pressure. J. Mol. Cell. Cardiol. 25:185–196. 8474126 10.1006/jmcc.1993.1021

[phy212815-bib-0031] Nergui, S. , Y. Fukumoto , E. Z. Do , S. Nakajima , T. Shimizu , S. Ikeda , et al. 2014 Role of endothelial nitric oxide synthase and collagen metabolism in right ventricular remodeling due to pulmonary hypertension. Circ. J. 78:1465–1474. 24705390 10.1253/circj.cj-13-1586

[phy212815-bib-0032] Nozawa, T. , Y. Yasumura , S. Futaki , N. Tanaka , M. Uenishi , and H. Suga . 1988 Efficiency of energy transfer from pressure‐volume area to external mechanical work increases with contractile state and decreases with afterload in the left ventricle of the anesthetized closed‐chest dog. Circulation 77:1116–1124. 3359589 10.1161/01.cir.77.5.1116

[phy212815-bib-0033] Ooi, C. Y. , Z. Wang , D. M. Tabima , J. C. Eickhoff , and N. C. Chesler . 2010 The role of collagen in extralobar pulmonary artery stiffening in response to hypoxia‐induced pulmonary hypertension. Am. J. Physiol. Heart Circ. Physiol. 299:H1823–H1831. 20852040 10.1152/ajpheart.00493.2009 PMC3006281

[phy212815-bib-0034] Otsuki, T. , S. Maeda , M. Iemitsu , Y. Saito , Y. Tanimura , R. Ajisaka , et al. 2006 Contribution of systemic arterial compliance and systemic vascular resistance to effective arterial elastance changes during exercise in humans. Acta Physiol. (Oxf) 188:15–20. 16911249 10.1111/j.1748-1716.2006.01596.x

[phy212815-bib-0035] Pacher, P. , T. Nagayama , P. Mukhopadhyay , S. Batkai , and D. A. Kass . 2008 Measurement of cardiac function using pressure‐volume conductance catheter technique in mice and rats. Nat. Protoc. 3:1422–1434. 18772869 10.1038/nprot.2008.138 PMC2597499

[phy212815-bib-0036] Pauschinger, M. , D. Knopf , S. Petschauer , A. Doerner , W. Poller , P. L. Schwimmbeck , et al. 1999 Dilated cardiomyopathy is associated with significant changes in collagen type I/III ratio. Circulation 99:2750–2756. 10351968 10.1161/01.cir.99.21.2750

[phy212815-bib-0037] Peterson, J. T. , H. Hallak , L. Johnson , H. Li , P. M. O'Brien , D. R. Sliskovic , et al. 2001 Matrix metalloproteinase inhibition attenuates left ventricular remodeling and dysfunction in a rat model of progressive heart failure. Circulation 103:2303–2309. 11342481 10.1161/01.cir.103.18.2303

[phy212815-bib-0038] Porterfield, J. E. , A. T. Kottam , K. Raghavan , D. Escobedo , J. T. Jenkins , E. R. Larson , et al. 2009 Dynamic correction for parallel conductance, GP, and gain factor, alpha, in invasive murine left ventricular volume measurements. J. Appl. Physiol. (1985) 107:1693–1703. 19696357 10.1152/japplphysiol.91322.2008 PMC2793194

[phy212815-bib-0039] Safdar, Z. , E. Tamez , W. Chan , B. Arya , Y. Ge , A. Deswal , et al. 2014 Circulating collagen biomarkers as indicators of disease severity in pulmonary arterial hypertension. JACC Heart Fail. 2:412–421. 25023820 10.1016/j.jchf.2014.03.013 PMC4180669

[phy212815-bib-0040] Sanz, J. , A. Garcia‐Alvarez , L. Fernandez‐Friera , A. Nair , J. G. Mirelis , S. T. Sawit , et al. 2012 Right ventriculo‐arterial coupling in pulmonary hypertension: a magnetic resonance study. Heart 98:238–243. 21917658 10.1136/heartjnl-2011-300462

[phy212815-bib-0041] Schreier, D. , T. Hacker , G. Song , and N. Chesler . 2013 The role of collagen synthesis in ventricular and vascular adaptation to hypoxic pulmonary hypertension. J. Biomech. Eng. 135:021018. 23445063 10.1115/1.4023480 PMC3705819

[phy212815-bib-0042] Tabima, D. M. , and N. C. Chesler . 2010 The effects of vasoactivity and hypoxic pulmonary hypertension on extralobar pulmonary artery biomechanics. J. Biomech. 43:1864–1869. 20416876 10.1016/j.jbiomech.2010.03.033 PMC2908025

[phy212815-bib-0043] Tabima, D. M. , T. A. Hacker , and N. C. Chesler . 2010 Measuring right ventricular function in the normal and hypertensive mouse hearts using admittance‐derived pressure‐volume loops. Am. J. Physiol. Heart Circ. Physiol. 299:H2069–H2075. 20935149 10.1152/ajpheart.00805.2010 PMC3006289

[phy212815-bib-0044] Tabima, D. M. , A. Roldan‐Alzate , Z. Wang , T. A. Hacker , R. C. Molthen , and N. C. Chesler . 2012 Persistent vascular collagen accumulation alters hemodynamic recovery from chronic hypoxia. J. Biomech. 45:799–804. 22183202 10.1016/j.jbiomech.2011.11.020 PMC3294039

[phy212815-bib-0045] Vanderpool, R. R. , and N. C. Chesler . 2011 Characterization of the isolated, ventilated, and instrumented mouse lung perfused with pulsatile flow. J. Vis. Exp. 50:2690. 21559007 10.3791/2690 PMC3197425

[phy212815-bib-0046] Wang, Z. , and N. C. Chesler . 2011 Pulmonary vascular wall stiffness: an important contributor to the increased right ventricular afterload with pulmonary hypertension. Pulm. Circ. 1:212–223. 22034607 10.4103/2045-8932.83453 PMC3198648

[phy212815-bib-0047] Wang, Z. , and N. C. Chesler . 2012 Role of collagen content and cross‐linking in large pulmonary arterial stiffening after chronic hypoxia. Biomech. Model. Mechanobiol. 11:279–289. 21538012 10.1007/s10237-011-0309-z PMC3248635

[phy212815-bib-0048] Wang, Z. , R. S. Lakes , J. C. Eickhoff , and N. C. Chesler . 2013a Effects of collagen deposition on passive and active mechanical properties of large pulmonary arteries in hypoxic pulmonary hypertension. Biomech. Model. Mechanobiol. 12:1115–1125. 23377784 10.1007/s10237-012-0467-7 PMC3745811

[phy212815-bib-0049] Wang, Z. , R. S. Lakes , M. Golob , J. C. Eickhoff , and N. C. Chesler . 2013b Changes in large pulmonary arterial viscoelasticity in chronic pulmonary hypertension. PLoS ONE 8:e78569. 24223157 10.1371/journal.pone.0078569 PMC3819365

[phy212815-bib-0050] Wang, Z. , D. A. Schreier , T. A. Hacker , and N. C. Chesler . 2013c Progressive right ventricular functional and structural changes in a mouse model of pulmonary arterial hypertension. Physiol. Rep. 1:e00184. 24744862 10.1002/phy2.184 PMC3970737

[phy212815-bib-0051] Wei, S. , L. T. Chow , I. O. Shum , L. Qin , and J. E. Sanderson . 1999 Left and right ventricular collagen type I/III ratios and remodeling post‐myocardial infarction. J. Card. Fail. 5:117–126. 10404351 10.1016/s1071-9164(99)90034-9

[phy212815-bib-0052] Wu, H. , M. H. Byrne , A. Stacey , M. B. Goldring , J. R. Birkhead , R. Jaenisch , et al. 1990 Generation of collagenase‐resistant collagen by site‐directed mutagenesis of murine pro alpha 1(I) collagen gene. Proc. Natl Acad. Sci. USA 87:5888–5892. 2165607 10.1073/pnas.87.15.5888 PMC54434

[phy212815-bib-0053] Yin, F. C. , H. A. Spurgeon , K. Rakusan , M. L. Weisfeldt , and E. G. Lakatta . 1982 Use of tibial length to quantify cardiac hypertrophy: application in the aging rat. Am. J. Physiol. 243:H941–H947. 6216817 10.1152/ajpheart.1982.243.6.H941

